# Injuries associated with mechanical chest compressions and active decompressions after out-of-hospital cardiac arrest: A subgroup analysis of non-survivors from a randomized study

**DOI:** 10.1016/j.resplu.2023.100362

**Published:** 2023-01-31

**Authors:** Polina Petrovich, Per Olav Berve, Borghild Barth-Heyerdahl Roald, Håvard Wahl Kongsgård, Arne Stray-Pedersen, Jo Kramer-Johansen, Lars Wik

**Affiliations:** a Norwegian National Advisory Unit on Prehospital Emergency Medicine, Oslo University Hospital, Oslo, Norway; b Institute of Clinical Medicine, Faculty of Medicine, University of Oslo, Norway; c Air Ambulance Department, Division of Prehospital Services, Oslo University Hospital, Oslo, Norway; d Department of Pathology, Oslo University Hospital, Norway; e Department of Forensic Sciences, Division of Laboratory Medicine, Oslo University Hospital, Norway

**Keywords:** CPR, Cardiopulmonary resuscitation, Autopsies, Cause of cardiac arrest, Skeletal injuries, Visceral injuries, Mechanical chest compression device

## Abstract

**Background:**

Both skeletal and visceral injuries are reported after cardiopulmonary resuscitation (CPR). This subgroup analysis of a randomized clinical study describes/compares autopsy documented injury patterns caused by two mechanical, piston-based chest compression devices: standard LUCAS® 2 (control) and LUCAS® 2 with active decompression (AD, intervention) in non-survivors with out-of-hospital cardiac arrest (CA).

**Method:**

We compared injuries documented by autopsies (medical/forensic) after control and intervention CPR based on written relatives consent to use patients’ data. The pathologists were blinded for the device used. The cause of CA and injuries reported were based on a prespecified study autopsy template. We used Pearson's chi-squared test and logistic regression analysis with an alpha level of 0.05.

**Results:**

221 patients were included in the main study (April 2015–April 2017) and 207 did not survive. Of these, 114 (55%, 64 control and 50 intervention) underwent medical (*N* = 73) or forensic (*N* = 41) autopsy. The cause of CA was cardiac 53%, respiratory 17%, overdose/intoxication 14%, ruptured aorta 10%, neurological 1%, and other 5%. There were no differences between control and intervention in the incidence of rib fractures (67% vs 72%; *p*-value = 0.58), or sternal fractures (44% vs 48%; *p*-value = 0.65), respectively. The most frequent non-skeletal complication was bleeding (26% of all patients) and intrathoracic was the most common location. Ten of the 114 patients had internal organ injuries, where lungs were most affected.

**Conclusion:**

In non-survivors of OHCA patients, the most frequent cause of cardiac arrest was cardiogenic. Skeletal and non-skeletal fractures/injuries were found in both control and intervention groups. Bleeding was the most common non-skeletal complication. Internal organ injuries were rare.

## Introduction

In the western world, 70% of all cardiac arrests (CA) occur due to ischemic cardiac disease, and 49/100 000 Europeans are treated for sudden out-of-hospital CA (OHCA) annually.^1^ High-quality chest compressions (uninterrupted and sufficient compression depth/rate) are important to maintain circulation and improve survival.^2^ The force needed to achieve sufficient chest compression depth can be high and may result in iatrogenic injuries.^3^ Systematic reviews with pooled analysis report that mechanical compressions tend to cause more injuries than manual compressions.^4^, ^5^

The active decompression (AD) technique of cardiopulmonary resuscitation (CPR) is performed manually by applying a suction cup on the chest and typically pulling it above the anatomic neutral level.^14^ The intrathoracic pressure decreases, which facilitates an increased cardiac preload and consequently increases the stroke volume expelled by the subsequent chest compression.^6^ The hemodynamic benefits of AD have been studied experimentally^15^ and clinically.^7^ CPR injury is an independent factor for 30-day mortality.^16^ Thus, it is of clinical interest to explore any differences in CPR related injury patterns when a new mode of mechanical chest compressions (AD) is evaluated. Specifically, the extra force of actively lifting the patient's chest constitutes an unusual mechanical challenge and might cause more injuries. In the present study we will explore the distribution of injuries generated by this technique.

The aim of this secondary study from a randomized clinical trial was to describe (autopsies) the cause of CA and compare injury patterns caused by two mechanical, piston-based chest compression devices LUCAS® 2 and LUCAS® 2 AD in patients with non-traumatic OHCA.^7^ Both devices compress the chest 5.3 cm. The only difference is that the AD device pulls the chest 3 cm above the anatomic neutral level of the chest. This distance is similar to chest rise when breathing. Therefore, we hypothesized no differences in injury patterns between the devices.

## Method

### Study population and design

The study was conducted in cooperation with the emergency medical service (EMS) of Oslo and Akershus (Oslo University Hospital, Division of Prehospital Services). Adult patients with OHCA treated by ambulance crews and the rapid response car in Oslo were prospectively included in this cluster randomized, un-blinded clinical trial from April 2015 through April 2017.^7^ Patients were included based on balanced inclusion periods into standard Lund University Cardiopulmonary Assist System, LUCAS® 2 (control) or LUCAS® 2 AD (intervention) (both Stryker Emergency Care, Lund, Sweden). All clusters were pre-randomized with variable time interval sizes (1–4 weeks) for the two-year study period. Based on information in a sealed envelope, a study coordinator placed either a control or an intervention device in the rapid response car.^7^

### Inclusion and exclusion criteria

Eligible patients were non-survivors included in the randomized study by Berve et al. comparing hemodynamic measurements between LUCAS® 2 and LUCAS® 2 AD during OHCA CPR.^7^ Of the eligible patients, we included patients undergoing a medical/or forensic autopsy after a non-traumatic OHCA where next of kin had consented to use of data and participation in the study. Exclusion criteria was when next of kin did not consent.

### Properties of LUCAS® 2 and LUCAS® 2 AD

Both LUCAS® 2 and LUCAS® 2 AD are battery or mains powered, Conformite Européenne (CE) marked, piston-based chest compression devices with a suction cup. The devices compress the patient’s chest at a frequency of 102 compressions min^−1^, to a depth of approximately 5.3 cm. Both devices pull the patient’s sternum with the help of a suction cup.^8^ The LUCAS® 2 pulls the piston back to the chests anatomic resting level with a force [Newton (N)] of 13 ± 2 N and the LUCAS® 2 AD pull the piston additionally up to 30 mm above this level with a maximum force of 89 ± 4 N. The patient’s chest height (anterior posterior diameter) and compression/decompression forces are measured electronically by the devices based on piston operational data (downloaded after the case).

### Research procedure

All patients who did not survive were eligible for a medical/or forensic autopsy. According to Norwegian legislation, any sudden unexpected death is considered unnatural. The police or a higher prosecution authority will generally request a forensic post-mortem examination. If the medical history may explain the sudden CA, or the police for any reason did not request a forensic examination, a medical autopsy is indicated with informed consent from the next of kin.

Within 24 hours of death being established, the next of kin was contacted by the principal investigator (or someone representing he/she). The circumstances of death and medical considerations were presented and the possibility of a medical/or forensic autopsy to document cause of CA and CPR related injuries were discussed. If a forensic autopsy was not to be carried out, a medical autopsy was requested by the rapid response car doctor if next of kin consented. We were only allowed to use data from either medical/or forensic autopsy reports when permission from the patients’ next of kin was granted by written consent. Later, a follow-up conversation was performed to share and explain the autopsy findings to the next of kin.

### Variables

The primary outcome variables were injuries after LUCAS® 2 compared to LUCAS® 2 AD CPR and cause of OHCA based on autopsy reports. The secondary outcome measurements were association between CPR duration, age, sex and injuries.

Duration of CPR was measured from start of mechanical chest compressions, verified electronically by reviewing LIFEPAK 15 (Stryker Emergency Care, Redmond, WA, USA) defibrillator files in CODE-STAT 10 data review software (Stryker Emergency Care, WA, USA). The LUCAS® devices electronically recorded force and depth data on each compression/decompression, chest height, and in addition duration of mechanical CPR. Manual CPR time prior to application of the mechanical chest compression device was calculated from patient report forms. CPR duration was registered continuously until (1) the patient was declared dead, or (2) the patient received sustained ROSC (a palpable pulse > 20 minutes). In-depth information can be found in Berve et al.^7^

### Data collection pathology

We provided a standardized autopsy protocol to the pathologist prior to study start. All cases were examined similarly, and the following assessments were included (1) skeletal injuries, (2) injuries on internal organs, (3) bleeding, and (4) cause of death. Cause of death was categorized into cardiac, respiratory, overdose/intoxication, bleedings, neurological, and other. Pathologists reported presence and localization of bleeding. Small soft tissue hemorrhage in the immediate vicinity of the fracture site were disregarded and not classified as bleeding. Hemorrhagic fluid (hematomas) in body cavities were estimated in liters (L). Truncal bleeding was classified into (1) mild/moderate soft tissue hemorrhage (retrosternal or intramuscular, amount in L was not described by pathologist), (2) significant intra-abdominal hemorrhage (>0.3 L), or (3) significant intrathoracic hemorrhage (large soft tissue hemorrhages or hematomas in pleura (>0.2 L), pericardium, and/or mediastinum (>0.1 L). The forensic post-mortem examination included similar registry of data, but also included full body computed tomography (CT) scan of all cases. Data was extracted from the autopsy reports to the study database.

### Statistical analysis

We performed statistical analyses using SPSS 26 (IBM, IL; USA). Pearson's chi-squared test was used for categorical variables. We considered *p*-values less than 0.05 as statistically significant. We did not perform any analysis on missing data. Multivariate logistic regression analysis was used to obtain odds ratio (OR) with 95% confidence intervals (CI) for the following outcomes: rib fractures, sternal fractures, non-skeletal injuries, and significant intrathoracic bleeding adjusted for sex, age (above or below 65 years), CPR duration (above or below median 38 minutes), and control vs intervention.

Power calculation for this study was performed on the primary outcome of the original study.^7^ We did not perform a priori power calculation for the secondary outcomes. A post-hoc power calculation for a difference in rates of post-CPR injuries with the current sample size in an unadjusted statistical test, reveals that a difference of at least 0.2 from baseline rate 0.7 for skeletal injuries, at least 0.2 from baseline rate 0.05 for non-skeletal injuries, and at least 0.25 from baseline rate 0.2 for any bleeding, could be falsified with a power (1–beta) of at least 80%.

### Ethical approval

The Regional Ethical Committee (REK 2014/1214, Norway) approved this study in 2015 and it was registered at ClinicalTrials.gov (NCT02479152).

## Results

In total, 221 patients with non-traumatic OHCA were accessed for eligibility (April 2015–April 2017), of whom 207 died (14survived). Of the 207 who died, we included 114 patients and excluded 93 patients (16 no consent and 77 no autopsy). Consent to use autopsy reports were obtained from next of kin.

Medical autopsy was performed on 73 (64%) patients and forensic autopsies on 41 (36%) patients. The LUCAS® 2 group consisted of 64 patients and the LUCAS® 2 AD of 50 patients (Table 1). The age and sex distribution were comparable, even after excluding patients with missing data.

**Table 1:** Demographics of non-survivors who underwent autopsy and description of cause of cardiac arrest for the two groups.

	All	LUCAS® 2	LUCAS® 2 AD	*p*-value
Study population	114	64	50	
Age (median (range))	64 (19–91)	62 (19–91)	66 (19–84)	0.93
Male, *n* (%)	79 (69%)	44 (69%)	35 (70%)	0.89*
Median anterior-posterior chest diameter in mm (Median (25-and 75 percentiles))	204 (184, 223)	209 (184, 225)	198 (181, 219)	0.42
Median compression depth in mm (Median (25-and 75 percentiles)), missing 2 and 5	51 (49, 52)	51 (50, 52)	51 (49, 52)	0.66
Median compression force in Newton (Median (25- and 75 percentiles) ), missing 2 and 4	460 (400, 530)	475 (400, 533)	440 (368, 520)	0.34
Median decompression in mm (Median (25- and 75 percentiles) ), missing 5			28 (24, 29)	na
Median decompression in Newton (Median (25- and 75 percentiles) ), missing 5			30 (9, 52)	na
Duration CPR (min)				
Manual (median (25- and 75 percentiles))	10 (6, 14)	10 (7, 14)	9 (5, 15)	0.51
Mechanical (median (25- and 75 percentiles))	25 (14, 36)	29 (15, 41)	21 (12, 32)	0.04
Total (median (25- and 75 percentiles))	36 (25, 47)	40 (26, 57)	32 (23, 40)	0.02
Type of autopsy				0.69*
Medical (*n*)	73 (64%)	42 (66%)	31 (62%)	
Forensic (*n*)	41 (36%)	22 (34%)	19 (38%)	
Cause of cardiac arrest (autopsy)				
Cardiac	60 (53%)	34 (53%)	26 (52%)	
Cardiogenic	14	4	10	
Myocardial infarction	36	23	13	
Heart tamponade	6	4	2	
Other cardiac	4	3	1	
Ruptured aorta	11 (10%)	7 (11%)	4 (8%)	
Thoracal aorta	9	5	4	
Abdominal aorta	2	2	0	
Respiratory	20 (17%)	12 (19%)	8 (16%)	
Foreign body	3	1	2	
Pulmonary embolus	8	6	2	
Infection	9	5	4	
Overdose/intoxication	16 (14%)	6 (9%)	10 (20%)	
Neurological	2 (1%)	1 (1%)	1 (2%)	
Other**	6 (5%)	5 (7%)	1 (2%)	

All *p*-values from independent samples Mann-Whitney U-test except those marked * which are from Chi-square test. For decompression statistics only data from LUCAS 2 AD (intervention group) is available, and statistical testing is not applicable (na). ** Other includes injury to spinal cord due to fall (*n* = 1), hyperglycemic (*n* = 1), and cause of death not classified by pathologist (*n* = 4).

The median age for the study population was 64 years (range: 19–91) and men represented 70% of all patients. The most common cause of CA based on autopsy reports was cardiac 53% (CA, myocardial infarction), followed by respiratory 17% (respiratory arrest, pulmonary embolus, pneumonia) and intoxications 14% (opioids, amphetamine), ruptured aorta 10%, neurological 1%, and other 5% (Table 1). In the control group, bleeding was considered causative of the CA in 7 (11%) cases, compared to 4 (8%) in the intervention group. The distribution of bleeding caused by CPR between control and intervention were related to pulmonary embolism (*n* = 1 vs *n* = 1), myocardial infarction (*n* = 3 vs *n* = 2), and cardiac (*n* = 3 vs *n* = 1), respectively.

In general, there were no significant associations between any of the injury patterns and the independent variables tested in unadjusted or adjusted models with one or two exceptions (Table 3). We found that female sex and age above median were both associated with increased odds ratio for significant intrathoracic bleedings (Table 3d).

**Table 2:** Skeletal and non-skeletal injuries of non-survivors described in autopsy reports for all included patients.

	All (*n* = 114)	LUCAS® 2 (*n* = 64)	LUCAS® 2 AD (*n* = 50)	*p*-value
Skeletal injuries				
Rib fractures (%)	79 (69%)	43 (67%)	36 (72%)	0.58
Number of rib fractures (median)	6	5	7	0.37*
Sternal fractures (%)	52 (46%)	28 (44%)	24 (48%)	0.65
Non-skeletal injuries	All	LUCAS® 2	LUCAS® 2 AD	*p*-value
Any injuries on internal organs	10 (9%)	3 (5%)	7 (14%)	0.10
Lung	6 (5%)	2 (3%)	4 (8%)	
Liver	2 (2%)	0	2 (4%)	
Spleen	1 (0.01%)	1 (2%)	0	
Diaphragm	1 (0.01%)	0	1 (2%)	
Bleeding	All	LUCAS® 2	LUCAS® 2 AD	*p*-value
Any type of bleeding	30 (26%)	15 (23%)	15 (30%)	0.43
Minor soft tissue (retrosternal + muscular)**	6 (5%)	3 (5%)	3 (6%)	
Significant intraabdominal***	4 (4%)	2 (3%)	2 (4%)	
Significant intrathoracic****	20 (18%)	10 (16%)	10 (20%)	

Number and percentage within each group with the characteristic.

L = litres.

AD = Active Decompression.

All *p*-values from Chi-square tests, except for number of rib fractures (marked with * and tests with independent samples Mann-Whitney U-test).

** Minor soft tissue bleeding classified as any parenchymal bleeding of non-significant amount. Not described in L by pathologists.

*** Significant intraabdominal bleeding classified as any bleeding found in the abdominal, and described with L by pathologists.

**** Significant intrathoracic bleeding classified as any bleeding found in the thoracic cavity, and described with L by pathologists.

Of those bleedings evaluated as significant (intrathoracic: pericardia > 0.1 L and/or pleural > 0.2 L, and abdominal > 0.3 L) there were 12 patients (19%) in the LUCAS 2 group and 11 (22%) in the LUCAS 2 AD group.

**Table 3:** Factors associated with injuries of non-survivors detected at autopsy.

	Unadjusted OR (95% CI)	*p*-value	Adjusted OR (95% Cl)	*p*-value
(a) Any rib fractures				
LUCAS® 2 AD vs LUCAS® 2 (ref)	1.26 (0.56–2.82)	0.58	1.15 (0.48–2.77)	0.75
Age (older than median)	0.98 (0.44–2.18)	0.96	1.04 (0.46–2.39)	0.92
Sex (female)	1.16 (0.48–2.77)	0.74	0.95 (0.36–2.47)	0.91
Chest height (AP-diameter larger than median)	1.37 (0.61–3.05)	0.44	1.49 (0.59–3.72)	0.40
Compression force (N) larger than median (missing 7)	1.47 (0.63–3.46)	0.38	1.28 (0.49–3.30)	0.61
Duration (Total), longer than median	1.22 (0.55–2.71)	0.63	1.03 (0.45–2.36)	0.95
(b) Sternal fracture				
LUCAS® 2 AD vs LUCAS® 2 (ref)	1.19 (0.57–2.49)	0.65	1.22 (0.53–2.80)	0.64
Age (older than median)	1.75 (0.83–3.70)	0.14	1.71 (0.77–3.79)	0.19
Sex (female)	0.85 (0.38–1.90)	0.69	0.89 (0.36–2.22)	0.80
Chest height (AP-diameter larger than median)	0.86 (0.41–1.79)	0.68	0.86 (0.36–2.06)	0.73
Compression force (N) larger than median (missing 7)	0.76 (0.35–1.68)	0.50	0.74 (0.31–1.81)	0.51
Duration (Total), longer than median	1.89 (0.90–3.98)	0.09	1.77 (0.80–3.94)	0.16
(c) Non-skeletal injuries				
LUCAS® 2 AD vs LUCAS® 2 (ref)	3.31 (0.81–13.53)	0.10	3.39 (0.74–15.44)	0.12
Age (older than median)	1.68 (0.45–6.32)	0.52	1.19 (0.29–4.81)	0.81
Sex (female)	1.57 (0.41–5.95)	0.49	1.88 (0.42–8.33)	0.41
Chest height (AP-diameter larger than median)	0.43 (0.11–1.75)	0.32	0.50 (0.10–2.42)	0.39
Compression force (N) larger than median (missing 7)	1.13 (0.30–4.27)	0.86	1.00 (0.21–4.77)	1.00
Duration (Total), longer than median	0.67 (0.18–2.50)	0.74	0.84 (0.21–3.34)	0.81
(d) Significant intra-thoracic bleeding				
LUCAS® 2 AD vs LUCAS® 2 (ref)	1.35 (0.51–3.55)	0.54	1.78 (0.57–5.56)	0.32
Age (older than median)	3.02 (1.07–8.53)	0.03	2.91 (0.95–8.95)	0.06
Sex (female)	2.76 (1.03–7.42)	0.04	4.41 (1.41–13.83)	0.01
Chest height (AP-diameter larger than median)	0.67 (0.25–1.78)	0.42	1.15 (0.35–3.77)	0.81
Compression force (N) larger than median (missing 7)	0.69 (0.23–1.91)	0.45	0.37 (0.10–1.40)	0.14
Duration (Total), longer than median	1.04 (0.40–2.74)	0.93	1.24 (0.43–3.60)	0.69

All independent variables were analyzed as categorical variables (intervention vs control (reference)), sex (female vs male (reference)), and age, chest height, applied chest compression force, and total duration of CPR: median or higher vs lower than median (reference). The adjusted model included all independent variables.

At least one rib fracture occurred in 79 patients (70%) with no difference between the two groups. The median number of fractured ribs per patient were 5 (control) vs 7 (intervention), *p* = 0.37. For 9 patients the number of fractured ribs was not specified by the pathologists (Table 2). Overall, sternal fractures were found in 52 patients (46%) with no difference between the standard and the intervention groups (Table 2).

Ten patients (9%) had internal organ injuries. Lacerations on lungs and liver were most common. There was a higher number of internal organ injuries in the intervention group, but this was not statistically significant (Table 2). The most frequent non-skeletal complication was bleeding, found in 30 (26%) patients, of these the most common location was significant intrathoracic bleedings (20 patients). There was no difference in the incidence of bleeding between the two groups (Table 2).

We performed four logistic regression analyses to ascertain the association between some independent variables on the occurrence of any rib fracture, sternal fracture, visceral injuries or significant intrathoracic hemorrhage (Table 3). In general, there were no significant associations between any of the injury patterns and the independent variables tested in unadjusted or adjusted models with one or two exceptions. We found that female sex and age above median were both associated with increased odds ratio for significant intrathoracic bleedings (Table 3d).

## Discussion

In this study of non-survivors after OHCA we describe and compare injury patterns caused by two mechanical, piston-based chest compression devices, the LUCAS® 2 and the LUCAS® 2 AD. The most frequent cause of CA was cardiac (53%).^1^ We found no statistically significant differences in rib fractures, sternal fractures, or non-skeletal injuries. Both rib and sternal fractures were common, 70% and 45%, respectively. The most frequent non-skeletal CPR related complication was bleeding which was found in 26% of all patients. These findings correspond to a previously published study where the patients received manual standard chest compressions or AD.^5^

Although this study documented the cause of cardiac arrest based on autopsies, it is comparable to the 60% of cardiac cause documented by the Norwegian Cardiac Arrest Registry 2021. Compared to the Registry data other causes like respiratory, intoxication, bleeding, and neurological were 17% vs 10%, 14% vs 8%, 10% vs 2%, 1% vs 8%, respectively. Differences in cause of arrest in our study and in the registry presumably have two main reasons. Firstly, selection bias in our study since it only includes patient that did not survive the cardiac arrest while the registry also includes surviving patients where a higher proportion will have reversible causes. Secondly, presumed cause of cardiac arrest in the Norwegian Cardiac Arrest Registry is not routinely adjudicated, and as such it is the best presumption of the treating personnel.

Two factors usually contribute to injuries. One is the force applied to the patient’s chest and the other is CPR duration. Patient sex, age, muscle mass, and previous medical conditions may be associated factors. Tomlinson et al. investigated the relationship between forces (kilograms) applied to the chest of OHCA patients with compression depth.^3^ The authors found that a stiffer chest required application of more force than a softer chest to achieve the same compression depth, and that there was large variation between patients. Skeletal injuries are often associated with older age and female sex.^8^, ^9^, ^10^, ^11^, ^12^ We may speculate that this is caused by osteoporosis. We did not find any association between applied forces on chest (Newton), age, or female sex with skeletal or visceral injuries (Table 3). In a OHCA study by Azeli Y et al. they determined the CPR compression force variation (CFV, the difference between the maximum and minimum force values of the first 30 minutes of LUCAS CPR) and its relationship with chest injuries and survival.^13^ The median number of rib fractures, sternum fractures, bilateral fractures were higher for high positive chest compression force variation (CFV, ≥95 N), and high negative CFV (<−95 N) compared to low CFV (<95 N). The variation was positive when the maximum force value was observed after the minimum value along the CPR duration and negative when maximum was observed before the minimum value.^13^

Non-skeletal injuries are relatively rare.^5^ In the present study 10% of the patients had injuries to internal organs. The pathologist documented significant bleeding in 12 (19%) LUCAS® 2 and 11 (22%) LUCAS® 2 AD patients. Generally, this is a high percentage, but it is the seriousness of the injuries that may have a clinical impact. In eight (13%) control group cases the bleedings were considered as contributing to the cause of death. In the intervention group, the corresponding number was four (8%). The pathologists described all injuries to internal organs to have little/no impact on patients’ outcome. Common locations were lung lacerations, injuries to the pericardium, or other mediastinum structures. This is expected due to the force applied to the thorax. A theoretical concern in our study was that AD could increase the incidence of bleeding. We did not find a difference between the two groups in our study. However, we documented that intrathoracic bleeding (Table 3) was associated with female sex and age above median. Severe injuries to the liver are potentially serious. The pathologist reported two such cases, but they were not considered as serious, which correspond with relevant literature.^17^ The role of manual chest compressions (performed by lay people, first responders and ambulance crews) before mechanical chest compressions as potential cause of CPR related injuries, is difficult to document. We did not measure the actual force or chest properties (stiffness) in our study. Devices measuring both force and depth could be applied during resuscitation and possibly refine analyses of chest compression dosage in relation to injuries.

In the study by Levy et al patients in both manual and mechanical CPR groups received manual compressions initially.^18^ As in our study, the patients treated with mechanical CPR are the patients that are “hard to resuscitate” and consequently, have received a “higher dose” of CPR. Levy et al. found that patients receiving only manual CPR (19patients) had a time-to-ROSC of median 3.3 (IQR 2.2–5.1) minutes. While the twenty-one patients (70%) with mechanical CPR, first received manual CPR for median 6.9 minutes (IQR 5.3–11), and then additional 11.2 (IQR 5.7–23.8) minutes of mechanical CPR before ROSC.^18^ Longer CPR duration may contribute to more injuries as documented by Krischer et al.^10^ Ondruschka et al. documented more injuries with longer mechanical chest compression time compared with manual.^19^ We also found that CPR duration > 38 minutes (median) increased the risk of significant intrathoracic bleedings (Table 3). Based on CT, Kim et al. did not document that CPR duration increased the number of skeletal injuries.^20^ A potential explanation may be inequalities in reporting the duration and how chest compressions were performed.

## Limitations

Bystanders and/or EMS personnel performed manual chest compressions before the deployment of a mechanical chest compression device. Injuries may have taken place early in the CPR process.^11^ This makes it difficult to conclude if it was manual or mechanical chest compressions that caused the injuries, or the combination of them. All our patients received manual chest compressions prior to randomized application of a mechanical compression device, making this concern less relevant for the comparisons in this study.

Few patients survived to hospital discharge due to the inclusion criteria. Patients with ROSC before rapid response car arrival were excluded. The overall survival rate in Oslo was 16% which is higher than the 6% we found in our study.^21^ This illustrates the limitation of reviewing outcome based on a study designed for hemodynamic measurements.

CT scans are more sensitive at detecting complications such as pneumothorax/pneumoperitoneum. Only the forensic autopsy patients received CT scanning prior to autopsy, which can explain why such complications were less commonly found during medical autopsies. Yamaguchi et al. suggest that simultaneous CT investigation in addition to autopsy shows significantly higher incident of injuries.^22^

The sample size was planned for the primary endpoint of the original study. The post-hoc power calculations reveal a risk for type II errors due to limited number of cases. Still, this is one of the larger consecutive studies of injuries after mechanical CPR. The primary study was randomized (block-randomized with variable block size). For this secondary study we did not include surviving patients, and not every patient that died was subject to autopsy. This introduces a risk for selection bias. We have no reason to believe that group assignment influenced decision or consent process for autopsy, and it certainly could not affect the decisions about forensic autopsies. In the logistic regression analyses (Table 3), the estimated association between non-skeletal injuries and the intervention device was high but with very wide confidence intervals. We cannot rule out that a higher number of autopsies could have verified such an association. The other associations between injury patterns and the intervention device were small and insignificant and did not change substantially when adjusted for other plausible independent variables.

## Conclusion

In non-surviving OHCA patients who received mechanical CPR most had a cardiac cause of arrest and skeletal injuries were common. Internal organ injuries were rare. Bleeding was the most common non-skeletal complication. Female gender and older age were associated with increased risk of intrathoracic bleeding. We conclude that mechanical chest compressions with active decompression do not cause more injuries than standard mechanical chest compressions.

## Funding

Norwegian National Advisory Unit on Prehospital Emergency Medicine (NAKOS), National association for Heart and Lung Patients (LHL), and Stryker (study coordinator).

## Conflict of Interest Statement

LW is a member of the Medical Advisory Board Stryker and hold patents through Oslo University Hospital Inven2 licensed to Stryker and Zoll. The other authors report no conflicts of interest.

## CRediT authorship contribution statement

**Polina Petrovich:** Conceptualization, Methodology, Software, Formal analysis, Investigation, Data curation, Writing – original draft. **Per Olav Berve:** Conceptualization, Methodology, Investigation, Data curation, Writing – review & editing. **Borghild Barth-Heyerdahl Roald:** Conceptualization, Methodology, Investigation, Data curation, Writing – review & editing, Supervision. **Håvard Wahl Kongsgård:** Methodology, Investigation, Data curation, Writing – review & editing. **Arne Stray-Pedersen:** Conceptualization, Methodology, Investigation, Data curation, Writing – review & editing, Supervision. **Jo Kramer-Johansen:** Conceptualization, Methodology, Investigation, Data curation, Writing – original draft, Supervision. **Lars Wik:** Conceptualization, Methodology, Investigation, Data curation, Writing – original draft, Supervision, Project administration.
